# A cohort study on risk factors of high-density lipoprotein cholesterol hypolipidemia among urban Chinese adults

**DOI:** 10.1186/s12944-021-01449-1

**Published:** 2021-02-22

**Authors:** Fang Tang, Jian Wang, Stephen Nicholas, Dongfu Qian, Rugang Liu

**Affiliations:** 1 Center for Big Data Research in Health and Medicine, The First Affiliated Hospital of Shandong First Medical University & Shandong Provincial Qianfoshan Hospital, Jinan, 250014 China; 2 Shandong Provincial Qianfoshan Hospital, Cheeloo College of Medicine, Shandong University, Jinan, 250014 China; 3 Dong Fureng Economic and Social Development School, Wuhan University, No.54 Dongsi Lishi Hutong, Beijing, 100010 Dongcheng District China; 4 Center for Health Economics and Management at School of Economics and Management, Wuhan University, 299 Bayi Road, Wuchang District, Wuhan, 430072 Hubei Province China; 5 Australian National Institute of Management and Commerce, 1 Central Avenue Australian Technology Park, Eveleigh, Sydney NSW 2015 Australia; 6 Guangdong Institute for International Strategies, Guangdong University of Foreign Studies, Baiyun Avenue North, Guangzhou, 510420 PR China; 7 School of Economics and School of Management, Tianjin Normal University, West Bin Shui Avenue, Tianjin, 300074 China; 8 Newcastle Business School, University of Newcastle, University Drive, Newcastle, NSW 2308 Australia; 9 School of Health Policy & Management, Nanjing Medical University, No. 101 Longmian Avenue, Jiangning District Nanjing, 211166 PR China; 10 Center for Global Health, Nanjing Medical University, No. 101 Longmian Avenue, Jiangning District Nanjing, 211166 PR China

**Keywords:** High-density lipoprotein cholesterol hypolipidemia, Cox regression model, Urban Han Chinese adult, Cohort study

## Abstract

**Background:**

High-density lipoprotein cholesterol (HDL-C) hypolipidemia, a major type of dyslipidemia, has been associated with many kinds of diseases, such as stroke, coronary heart disease, obesity and diabetes, and has displayed an increasing prevalence in China. This study explores the risk factors of HDL-C hypolipidemia and makes recommendations for controlling and preventing HDL-C hypolipidemia and the diseases caused by it.

**Methods:**

Using a retrospective cohort study design, 26,863 urban adults without dyslipidemia, diabetes, cardiovascular and cerebrovascular diseases, hepatosis, renal insufficiency and thyroid diseases were enrolled in the study between 2010 and 2015. Data on each individual were collected at the 2010 baseline year and at a follow-up medical check. A Cox regression model was constructed to evaluate the influence of potential risk factors on the outcome event- HDL-C hypolipidemia.

**Results:**

The incidence of HDL-C hypolipidemia was 5.7% (1531/26863). Sex, age, body mass index (BMI), HDL-C, triglyceride (TG) and urea nitrogen (UN) were significant risk factors of HDL-C hypolipidemia. Men were more likely to develop HDL-C hypolipidemia than women during follow-up medical checks (HR = 1.258, *P* = 0.014). The incidence of HDL-C hypolipidemia in the over 65 years old group was higher than that of the ≤65 age group (HR = 1.276, *P* = 0.009). The incidence of HDL-C hypolipidemia increased with increasing BMI (HR = 1.030, *P* = 0.002), TG (HR = 1.321, *P* = 0.001) and UN (HR = 1.054, *P* = 0.019), while falling with increasing HDL-C in the baseline year (HR = 0.002, *P* < 0.001).

**Conclusions:**

Men, aged over 65, with high BMI were at the highest risk of developing HDL-C hypolipidemia. Measures should be taken to prevent HDL-C hypolipidemia even for healthy urban adults whose blood biochemical indicators were in the normal range when their level of TG, UN and HDL-C are closed to the border of the normal value range.

## Introduction

Over the last 30 years, the incidence of dyslipidemia, or abnormal amount of lipids, such as triglycerides, cholesterol and fat phospholipids, in the blood has increased gradually in the Chinese population [1]. Opoku et al. [2] found the age- and sex-standardized prevalence of dyslipidemia was 43% for the Chinese population, with women having higher prevalence of dyslipidemia than men in both urban (54.0% vs. 46.0%) and rural (52.0% vs. 48.0%) locations. High-density lipoprotein cholesterol (HDL-C) hypolipidemia, a major dyslipidemia, had a prevalence as high as one-third of the Chinese population in 2012 [3]. Previous research has shown that HDL-C hypolipidemia was strongly associated with stroke, coronary heart disease, obesity and diabetes [4–7], while higher levels of HDL-C was a protective factor of coronary heart disease and stroke [8].

HDL-C has usually been taken as an independent variable, with previous research on cardiovascular disease, kidney disease and metabolic syndrome [5, 7, 9–31], treating the ratio of triglyceride (TG) to HDL-C as an important factor to predict related diseases, such as hyperuricemia, long-term mortality, periodontal disease, and major adverse cardiovascular events (MACEs) [4, 32–39]. By identifying the risk factors of HDL-C hypolipidemia, the related diseases can be prevented and controlled. When taken as a dependent variable, previous studies have usually studied HDL-C hypolipidemia as part of dyslipidemia, rather than as a dependent variable itself [40–44]. Bi et al. [45] used ultrasound measurements of abdominal fat thickness to predict low HDL-C levels among Singaporean adult men and women. Drawing on the 2017 Korean National Health and Nutrition Examination Survey, Cho et al. [46] found that the HDL-C level showed a weak negative correlation with blood pressure and age, with women showing a sharper decrease in HDL-C with the increase in systolic blood pressure (SBP) and age than men. Also using Korean data, Cho et al. [47] found that the lowest income group showed a larger prevalence of low-HDL-C levels, and the male group showed a relatively mild decrease in the HDL-C level after mid-life. Based on Spanish data, Millán-Núñez et al. [48] found that being females, having an elevated body mass index (BMI), using tobacco, suffering diabetes mellitus, displaying low-alcohol consumption and having a low exercise rate were significantly associated with low HDL-C. From a study of Sri Lankan patients, Weerarathna et al. [49] revealed females, younger age, higher BMI and low-density lipoprotein cholesterol level over 100 mg/dL had significant associations with suboptimal HDL among patients with diabetes mellitus.

A major constraint of most of above studies has been the use of cross-sectional data. Since the time when HDL-C hypolipidemia was diagnosed was unknown, time was not considered as an important confounding factor influencing the development of the disease. In cross-sectional studies, all variables are measured at the same point in time, with no sequential order between independent variables and dependent variables. As a result, when there is a correlation between the independent and dependent variables, associations can be inferred, rather than causal relationships tested. By building a retrospective cohort database, the paper addresses this disadvantage, allowing causal conclusions to be drawn. Based on medical check-up data from Shandong, China, the paper analyzes the risk factors of HDL-C hypolipidemia among urban Han Chinese adults. Recommendations for controlling and preventing HDL-C hypolipidemia and its related diseases are also made.

## Methods

### Data source and sample

Data were obtained from the medical records of a routine health check-up program at the Centre for Health Management of the First Affiliated Hospital of Shandong First Medical University. Among the routine health check-up program, participants were asked about their medical history and abdominal ultrasound, thyroid ultrasound, electrocardiogram (ECG), electroencephalogram (EEG) or cardiac ultrasound performed, as well as laboratory analyses, including blood lipid, fasting blood glucose (FBG), liver function and renal function tests, undertaken. Based on those examination reports, the participants’ health status were diagnosed by the physician and recorded on the health examination report.

The inclusion criteria for this study population were urban Han participants aged 18 and above, with records of at least two health check-ups over more than 1 year from January 2010 to December 2015 and the exclusion criteria were participants with dyslipidemia, diabetes, cardiovascular and cerebrovascular diseases, hepatosis, renal insufficiency and thyroid diseases diagnosed on their health examination report. Dyslipidemia was diagnosed as TG ≥ 2.3 mmol/L (≥200 mg/dl), and/ or low-density lipoprotein cholesterol (LDL-C) ≥ 4.1 mmol/L (≥160 mg/dl), and/or total cholesterol (TC) ≥ 6.2 mmol/L (≥240 mg/dl), and/ or HDL-C ≤ 1.0 mmol/L (≤40 mg/dl) and/ or self-reported clinically diagnosed dyslipidemia. Diabetes was diagnosed as FBG ≥ 7.0 mmol/L (126 mg/dl) and/ or self-reported clinically diagnosed diabetes. Cardiovascular and cerebrovascular diseases, hepatosis, renal insufficiency and thyroid diseases were also assessed by physician based on the corresponding diagnostic criteria. This cohort study comprised 26,863 urban Han adults.

### Outcome event

The outcome event was HDL-C hypolipdemia. It was defined as HDL-C lower than 1.0 mmol/L during a follow-up examination according to 2016 Chinese guidelines for the management of dyslipidemia in adults [1].

### Independent variables

According to previous research, and constrained by the structure of health check-up records, data collected included sex, age, body mass index (BMI), blood pressure, FBG, blood lipid indices (HDL-C, TG, LDL-C), liver function indices (glutamic-pyruvic transaminase (ALT), glutamic oxalacetic transaminase (AST), γ-glutamyl transpeptidase (γ-GT)) and renal function indices (creatinine (Cr), urea nitrogen (UN), and uric acid (UA)). Smoking (yes/no) and drinking (yes/no) behaviors were included as covariates.

### Measurement of variables

The hospital collected all medical check-up data under standardized procedures. Participants were required to wear light clothes and no shoes when their height and weight were measured. Nurses calculated BMI (weight (kg) divided by squared height (m^2^)) and measured systolic blood pressure (SBP) and diastolic blood pressure (DBP) on the right arm of seated participants using Omron HEM-907 by the cuff-oscillometric method. Participants were required to fast at least 12 h before their venous blood samples were drawn, with FBG, blood lipid indices (HDL-C, TG, LDL-C), liver function indices (ALT, AST, γ-GT) and renal function indices (Cr, UN, and UA) measured by the laboratory specialists using standard clinical and laboratory protocols in the national accredited laboratory of the First Affiliated Hospital of Shandong First Medical University. HDL-C was measured by the testing system Roche Cobase c701 using the direct method-catalase scavenging method. The respondents were divided into the ≤65 age group and > 65 age group.

### Statistical analysis

Median (p50) and interquartile boundary values (p25, p75) were used to describe the continuous variables and percentages for categorical variables. The baseline characteristics between men and women were compared using Wilcoxon Rank-Sum (WRS) test for continuous variables and the chi-square test for categorical variables. Kaplan-Meier survival estimates and log-rank tests were used to analyze the differences of incidence density by sex and age. A Cox proportional hazards regression model was constructed to evaluate the influence of potential risk factors on HDL-C hypolipidemia, the outcome variable, diagnosed during follow-up health checks. First, independent variables were entered into the Cox regression model individually to explore the relationship between each independent variable and the outcome event. Next, a multiple Cox regression model was constructed, involving all independent variables that were significantly related to outcome event. All statistical analyses were performed using STATA SE, version 12.0.

## Results

### Baseline characteristics of respondents

Table 1 displays the characteristics of the 26,863 surveyed respondents in the baseline year (2010). Men comprised 53.27% of the respondents, with a median age of 41 (p25, p75: 32, 54), median height of 174 cm (p25, p75: 170, 178), median BMI of 24.49 (p25, p75: 22.45, 26.57); and women had a median age of 38 (p25, p75: 31, 47), median height of 163 cm (p25, p75: 160, 167), average BMI of 21.96 (p25, p75: 20.08, 24.13). There were significant differences between men and women across all variables (*P* < 0.001). Wilcoxon Rank-Sum and chi-square tests showed that all indicators including population characteristics, somatotype, blood pressure, blood glues, blood fat, liver function and renal function of men were higher than women, except HDL-C (*P* < 0.001).

**Table 1:** Baseline characteristics of respondents

Variables		Total	Male	Female	***Z/***χ^**2**^	***P***
		M(p25, p75) /N (%)	M(p25, p75) /N (%)	M(p25, p75) /N (%)		
Demographic characteristics	Age (year)	40 (31, 50)	41 (32, 54)	38 (31, 47)	17.61	< 0.001
	Age group(> 65y)	2140 (7.97)	1575(11.01)	565 (4.5)	386.12	< 0.001
Somatotype	Height(cm)	169 (163, 175)	174 (170, 178)	163 (160, 167)	116.34	< 0.001
	Weight(Kg)	66.3 (58, 76)	74 (67, 81.5)	58.5 (53.5, 64.5)	105.7	< 0.001
	BMI(Kg/m^2)	23.34 (21.07, 25.68)	24.49 (22.45, 26.57)	21.96 (20.08, 24.13)	58.61	< 0.001
Blood pressure	SBP(mmHg)	122 (112, 134)	127 (118, 138)	117 (108, 129)	47.58	< 0.001
	DBP(mmHg)	78 (70, 85)	81 (75, 89)	73 (66, 80)	62.69	< 0.001
Blood glucose	FBG(mmol/L)	5.05 (4.76, 5.38)	5.14 (4.84, 5.48)	4.96 (4.69, 5.26)	31.24	< 0.001
Blood fat	HDL-C(mmol/L)	1.51 (1.31, 1.75)	1.42 (1.25, 1.64)	1.62 (1.41, 1.85)	−45.8	< 0.001
	TG(mmol/L)	0.92 (0.69, 1.22)	1.03 (0.79, 1.32)	0.8 (0.61, 1.07)	47.12	< 0.001
	LDL-C(mmol/L)	2.62 (2.21, 3.03)	2.71 (2.31, 3.09)	2.51 (2.11, 2.94)	23.64	< 0.001
Liver function	ALT(U/L)	15.8 (11.9, 21.9)	18.9 (14.4, 25.7)	13 (10.2, 17)	66.73	< 0.001
	AST(U/L)	17.8 (14.8, 21.3)	18.9 (16, 22.7)	16.4 (13.8, 19.5)	43.27	< 0.001
	γ- GT(U/L)	16.8 (12.1, 25)	22 (16.4, 31.4)	12.7 (10.1, 16.6)	86.03	< 0.001
Renal function	Cr(umol/L)	67.6 (57.4, 78.4)	77.3 (70.5, 84.3)	57.1 (51.9, 62.7)	122.45	< 0.001
	UN(mmol/L)	4.7 (4, 5.6)	5.1 (4.4, 6)	4.3 (3.6, 5)	59.71	< 0.001
	UA(umol/L)	299.6 (247.5, 357.7)	346.6 (303.7, 392.6)	250.7 (217.7, 287)	103.64	< 0.001

### Survival time and incidence density

**Fig. 1:**
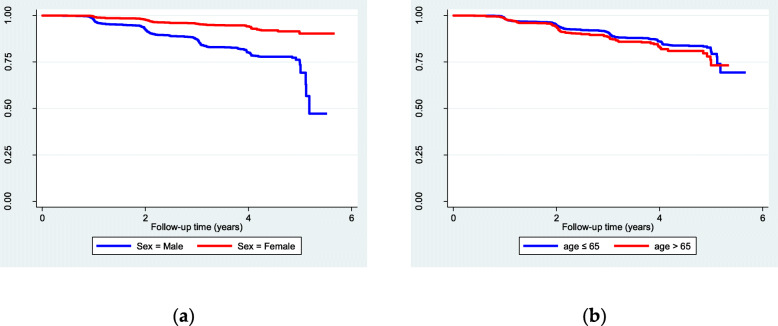
**a** Kaplan-Meier survival estimates (sex); **b** Kaplan-Meier survival estimates (age)

The incidence of HDL-C hypolipidemia was 5.7% (1531/26863), and the total incidence density of HDL-C hypolipidemia was 29.3/1000 person-years. Kaplan-Meier survival estimates in Fig. 1 and log-rank test in Table 2, show that the mean survival time for males was significantly smaller than females, the > 65 age group survival time was smaller than the ≤65 age group, and the incidence of male and the > 65 age group was significantly higher than the reference group (log-rank test, *P* < 0.05).

**Table 2:** Survival time and incidence density

Variables		Mean of survival time	SE	Incidence (%)	χ2	***P***
Sex	Male	4.73	0.05	8.73	386.2	< 0.001
	Female	5.43	0.02	2.25		
Age	≤65 group	5.09	0.03	5.57	6.42	0.011
	> 65 group	4.78	0.05	7.24		

### Univariate cox regression model

Single factor Cox regression models were constructed to analyze the relationship between the outcome event and each independent variable. Table 3 shows that there was a statistical correlation between HDL-C hypolipidemia and the independent variables, including sex, age, BMI, blood pressure factor, FBG, HDL-C, TG, LDL-C, ALT, γ- GT, Cr, UN, UA (*P* ≤ 0.01), and marginally significantly correlated with AST (*P* = 0.056 < 0.1). The HDL-C hypolipidemia incidence of males in the > 65 age group was higher than the ≤65 age group. HDL-C hypolipidemia incidence increased with increasing BMI, blood pressure, blood glucose, liver function indicators, renal function indicators and TG and LDL-C blood fat indicators, but dropped with increasing HDL-C.

**Table 3:** Results of single factor Cox regression

Variables(Reference)		β	SE	z	***P***	HR (95%CI)
Population characteristics	Sex(Female)	1.2204	0.0660	18.48	< 0.001	3.388(2.977,3.857)
	Age(≤65 group)	0.2143	0.0848	2.53	0.011	1.239(1.049,1.463)
Somatotype	BMI	0.1499	0.0068	21.92	< 0.001	1.162(1.146,1.177)
Blood pressure	Blood pressure factor	0.2547	0.0275	9.28	< 0.001	1.29(1.223,1.361)
Blood glucose	FBG	0.2002	0.0377	5.31	< 0.001	1.222(1.135,1.315)
Blood fat	HDL-C	−6.6591	0.1505	− 44.24	< 0.001	0.001(0.001,0.002)
	TG	1.6396	0.0714	22.98	< 0.001	5.153(4.481,5.927)
	LDL-C	0.5210	0.0475	10.97	< 0.001	1.684(1.534,1.848)
Liver function	ALT	0.0058	0.0006	10.35	< 0.001	1.006 (1.005,1.007)
	AST	0.0047	0.0025	1.91	0.056	1.005 (1.000,1.010)
	γ- GT	0.0033	0.0005	6.65	< 0.001	1.003 (1.002,1.004)
Renal function	Cr	0.0055	0.0005	11.20	< 0.001	1.005 (1.005,1.006)
	UN	0.1154	0.0189	6.10	< 0.001	1.122 (1.081,1.165)
	UA	0.0054	0.0003	20.35	< 0.001	1.005 (1.005,1.006)

### Multivariate cox regression model

All independent variables related to the outcome event (*P* < 0.1 in single factor Cox regression) were entered into a Cox regression model. As shown in Table 4, sex, age, BMI, HDL-C, TG and UN were significant risk factors for the development of HDL-C hypolipidemia. Men were more likely to develop HDL-C hypolipidemia than women during follow-up medical checks among urban adults (HR = 1.258, *P* = 0.014). The incidence of HDL-C hypolipidemia in the > 65 age group was higher than that of the ≤65 age group (HR = 1.276, *P* = 0.009); and the incidence of HDL-C hypolipidemia increased with increases in BMI (HR = 1.030, *P* = 0.002), TG (HR = 1.321, *P* = 0.001) and UN (HR = 1.054, *P* = 0.019) and decreased with HDL-C increasing in baseline year (HR = 0.002, *P* < 0.001).

**Table 4:** Results of multiple Cox regression

Variables* (Reference)		β	SE	z	***P***	HR (95%CI)
Population characteristics	Sex(Female)	0.229	0.094	2.45	**0.014**	1.258 (1.047,1.511)
	Age(≤65 group)	0.244	0.093	2.62	**0.009**	1.276 (1.063,1.532)
Somatotype	BMI	0.030	0.010	3.10	**0.002**	1.030 (1.011,1.050)
Blood pressure	Blood pressure factor	−0.021	0.036	−0.59	0.557	0.979 (0.912,1.051)
Blood glucose	FBG	0.005	0.045	0.11	0.911	1.005 (0.920,1.098)
Blood fat	HDL-C	−6.310	0.163	−38.66	**< 0.001**	0.002 (0.001,0.003)
	TG	0.278	0.085	3.25	**0.001**	1.321 (1.117,1.561)
	LDL-C	0.098	0.055	1.78	0.075	1.103 (0.990,1.228)
Liver function	ALT	0.004	0.003	1.35	0.177	1.004 (0.998,1.009)
	AST	−0.004	0.006	− 0.74	0.461	0.996 (0.985,1.007)
	γ- GT	0.000	0.002	0.15	0.880	1.000 (0.997,1.004)
Renal function	Cr	−0.005	0.002	−1.94	0.053	0.995 (0.990,1.000)
	UN	0.052	0.022	2.34	**0.019**	1.054 (1.009,1.101)
	UA	0.001	0.000	1.74	0.082	1.001 (1.000,1.001)

*Smoking and drinking behaviors were included as covariates

## Discussion

The present study explored the risk factors for the development of HDL-C hypolipidemia among urban Han Chinese adults. Sex, age, BMI, HDL-C, TG and UN in the baseline year were significantly associated with the development of HDL-C hypolipidemia during follow-up medical checks. The incidence of HDL-C hypolipidemia was 5.7% during follow-up medical checks, which was lower than other studies, where the prevalence percentage varied between 33.9 and 60.86% [1, 48, 50]. One reason was that previous samples comprised the entire population, while patients with metabolic diseases in this study were screened out in the baseline year. Males were 1.258 times as likely to have HDL-C hypolipidemia as females, which is different from the results of cross-sectional studies [46–50]. There are two possible reasons. First, participants in this study were different from those in other studies. Patients with metabolic diseases were screened out in the baseline year, while previous studies contained the whole population. Women’s morbidity of HDL-C hypolipidemia may be higher than men in the whole population at one point-in-time in cross-sectional studies. The second difference is due to the statistical methods. Using the survival analysis method, the likelihood of developing the disease over time was explored based on cohort data while previous studies described the current situation of HDL-C hypolipidemia morbidity at a specific period of time. The difference between men and women in this study may be due to their different basic physical situation [49].

The present study showed that the over 65 year old group was more likely to suffer HDL-C hypolipidemia than the younger under 65 age group. Age differences in the prevalence of HDL-C hypolipidemia has been reported in other studies [1]. In a study of diabetes patients with the prevalence of low HDL cholesterol and its associations, Weerarathna et al. [49] did not find any age difference in the prevalence of HDL-C hypolipidemia. The heterogeneity on age needs to be further studied physiologically. Depending on the outcome of further studies, males aged over 65 might be an intervention target for the management of HDL-C hypolipidemia.

In regard to obesity, measured by BMI, HDL-C hypolipidemia in this study increased as BMI increased, which is similar to previous studies [48, 49]. Keeping BMI as low as possible in the normal range is an effective way to prevent or attenuate the development of HDL-C hypolipidemia. Consistent with the present results, Cho et al. [46] also found an association between declining serum HDL-C and blood pressure and HDL-C hypolipidemia. This study also illustrated that HDL-C level in the baseline year had the greatest impact on the incidence of HDL-C hypolipidemia among all biological indicators, with HR only equal to 0.002. Higher levels of HDL-C in the baseline year could protect people from HDL-C hypolipidemia.

TG was the second strongly correlated variable with HDL-C hypolipidemia, followed by UN. The elevated level of TG and UN, and the decreased level of HDL-C in the baseline year, indicated an increased incidence of HDL-C hypolipidemia. Previous research has shown that it is essential to monitor lipid levels and to take interventions as early as possible to prevent dyslipidemia and its related complications [40]. The practical value of this study is that medical practitioners should not only focus on whether these indicators are within normal range or not, but also whether these indicators are close to the edge of the normal range. HDL-C hypolipidemia should not only be treated when it is diagnosed, but also preventive measures should be implemented when HDL-C hypolipidemia is on the margins of the normal values. Even for healthy people whose indicators are in the normal range, they should be advised to take measures to prevent HDL-C hypolipidemia when their indicators deviate from average levels.

### Comparisons with other urban areas in China

Low HDL-C was treated as a part of dyslipidemia in most past research in China, rather than as an independent outcome [40, 51]. While a few studies explored the risk factors of low HDL-C, they focused on comparing the difference between rural and urban residents [52, 53]. Most previous studies used cross-sectional data, with the prevalence of HDL-C hypolipidemia of Chinese urban residents, estimated at 20.8–28.4%, and correlations between risk factors and low HDL-C reported [2, 52–54]. One contribution of this cohort study meant that the results comprised both the incidence and the causality of HDL-C hypolipidemia.

## Study strengths and limitations

There are three major strengths in this study. First, this study built a cohort without metabolic diseases in the baseline year. This reduced the influence from other related diseases. Second, survival and cohort analysis methods were used to explore casual relationships, rather than correlations, between the dependent and independent variables. Third, more potential biochemical indicators were involved in the model.

This study has several limitations. First, lifestyle factors, such as diet and exercise habits, that may be associated with the occurrence of HDL-C hypolipidemia were not included in the dataset. Second, the short follow-up time period and low incidence density may affect the accuracy of Cox regression model. Third, the sample did not include rural residents where the incidence of dyslipidemia shows a tendency to rise more quickly than among urban residents. Finally, genetic information and family history related to cardiometabolic disorders were not included. These limitations might impose a modest constraint on the interpretation of these findings, but they should not substantively undermine the internal validity of the study.

## Conclusions

Sex, age, BMI, HDL-C, TG and UN were significant risk factors in the development of HDL-C hypolipidemia. Men, aged over 65 years old, with higher BMI, were at a higher risk of developing HDL-C hypolipidemia than women younger than 65 years old and with lower BMI. Health management strategies should be taken to prevent HDL-C hypolipidemia, even for healthy urban adults whose blood biochemical indicators were in the normal range, when the level of TG, UN and HDL-C were closed to the border of the normal value range.

## Data Availability

The datasets generated and/or analysed during the current study are not publicly available due to cooperation with the First Affiliated Hospital of Shandong First Medical University. The corresponding author will facilitate a discussion with First Affiliated Hospital of Shandong First Medical University for data access on a reasonable request.
